# Endocrine Toxicities of Antineoplastic Therapy

**DOI:** 10.3390/cancers13020294

**Published:** 2021-01-14

**Authors:** Giulia Puliani, Marialuisa Appetecchia

**Affiliations:** 1 Oncological Endocrinology Unit, IRCCS Regina Elena National Cancer Institute, 00144 Rome, Italy; giulia.puliani@ifo.gov.it; 2 Department of Experimental Medicine, Sapienza University of Rome, 00161 Rome, Italy

In recent years, the prognosis of many solid tumors has improved markedly thanks to new treatment strategies, including tyrosine kinase inhibitors (TKIs) and immunotherapy [1]. Despite their clinical relevance, these drugs are burdened with side effects, including endocrine ones, which can become serious and life-threatening if not promptly diagnosed and correctly managed [2]. TKIs act by inhibiting the catalytic activity of many kinases, consequently blocking multiple pathways linked to cell proliferation and survival, angiogenesis, and tumor invasion [3]. Thyroid dysfunctions during TKI therapies are very common, with an estimated incidence of 33.2% [4]. In patients with previous hypothyroidism, initiation of TKI therapy may require an increase in the levothyroxine dose. In patients without prior thyroid dysfunction, the most common side effect is the development of hypothyroidism possibly preceded by thyrotoxicosis due to destructive thyroiditis [5]. The pathogenesis is multifactorial and includes both direct thyroid damage, through the reduction in vascularity, inhibition of thyroid peroxidase activity, and onset of destructive thyroiditis, and peripheral effects, such as increased metabolism and clearance of thyroid hormones [5,6]. Thyroid toxicity has been proposed as a predictor of tumor response to TKIs, predicting longer progression-free survival [4,7].

The endocrine toxicity of TKIs goes beyond thyroid disorders and can also be characterized by metabolic complications, altered electrolyte and bone metabolism, and gonadal impairment. Metabolic complications are frequent when using TKIs, as dyslipidemia is reported in approximately 50% of treated patients and diabetes mellitus is reported in 15–40% of cases [8,9]. Worsening or onset of dyslipidemia has been associated with nearly all TKIs, with the exception of imatinib [10]. TKIs can reduce insulin sensitivity, inducing hyperglycemia [11]. Nilotinib appears to be the TKI with the greatest diabetogenic effect. However, it should be considered that TKIs can also cause hypoglycemia and improvement of previous diabetes mellitus [8].

Similar to TKIs, mammalian target of rapamycin (mTOR) inhibitors are also frequently associated with metabolic complications, such as dyslipidemia (both hypertriglyceridemia and hypercholesterolemia) and diabetes mellitus [12].

Both TKIs and mTOR inhibitors have been associated with electrolyte imbalances. The most common alteration is hyponatremia, due to the syndrome of inappropriate antidiuretic hormone secretion (SIADH) [13]. However, other imbalances, such as magnesium and potassium losses, can also occur if EGFR (epidermal growth factor receptor) is blocked [13].

TKIs can also have an impact on bone metabolism; in fact, many TKIs have been associated with secondary hyperparathyroidism and decreased bone turnover [14]. These alterations in bone turnover could be explained by the inhibition of kinases, such as c-KIT and platelet-derived growth factor receptor A, in osteoclasts and osteoblasts [15].

Immunotherapy aims to stimulate the immune response against neoantigens on the cell surface of cancer cells [16], as tumor cells have been shown to escape immune response by exploiting the inhibitory physiological pathways of programmed cell death protein 1 (PD-1) and its ligand (PD-L1) or cytotoxic T-lymphocyte antigen 4 (CTLA-4) [17]. These drugs have been approved for many types of cancer, but the landscape is constantly expanding with new targets and new indications [1,18]. Taking into account the ability of this class of antineoplastic drugs to enhance the immune response, it is easy to understand the high risk of immune-related adverse events associated with these treatments [19]. In particular, the dysregulation of the immune system and autoimmunity are responsible for many endocrine toxicities.

Thyroid dysfunctions are the most common side effects of immunotherapy, affecting 30% of treated patients. Hypothyroidism is more common in patients treated with PD-1 inhibitors and in combination therapies [20], and although adverse events are usually immune-mediated, it is caused by autoimmune thyroiditis in only 45% of cases [21]. PD-1 inhibitors are also associated with an increased risk of hyperthyroidism, which is usually transient and followed by hypothyroidism, although cases of Graves’ disease and thyroid eye disease have also been described [21].

Contrary to thyroid changes, hypophysitis is more associated with CTLA-4 inhibitors, but again, combination therapy increases the risk [22]. Patients may have both a bulking effect, due to increased pituitary tissue, and hormonal insufficiency: isolated adrenocorticotropic hormone (ACTH) deficiency is more common in patients treated with PD-1 and PD-L1, while CTLA-4 inhibitors usually cause multiple anterior pituitary insufficiency [21]. The concomitant development of diabetes insipidus and anterior pituitary deficiency due to CTLA-4 inhibitors has been rarely described. The development of secondary hypocortisolism could be life-threatening and requires immediate diagnosis and replacement therapy [23]. More rarely, immunotherapy has been associated with primary adrenal insufficiency (0.7% of patients) [20]. While rare, it is important to consider this potential side effect, as it could present with an adrenal crisis [21]. Finally, new onset or worsening of hyperglycemia has been described in up to 2% of patients, rarely associated with diabetic ketoacidosis requiring immediate treatment [21].

## 1. Old Therapies, New Concerns

Although the interest in endocrine toxicities of antineoplastic therapy has grown after the diffusion of target therapies and immunotherapy, “classical” chemotherapy is not free of endocrine side effects.

The most common complication in premenopausal women is menstrual cycle interruption due to many chemotherapy regimens [24]. Moreover, the toxic effects of chemotherapy on fertility are not always reversible, and the cryopreservation of oocytes for women and of spermatozoa for men should nowadays be part of the treatment strategies of these patients [25]. Sexual dysfunction may be a long-term complication of many cancer treatments, not only in case of direct impact on the pelvic region as for gynecologic cancer or prostate cancer, but also because of the systemic effects of chemotherapy [26,27]. Moreover, in patients affected by breast or prostate cancer, hormonal therapy induces bone loss altering physiological bone turnover and exposing patients to high risk of osteoporosis and fragility fractures [28]. Chemotherapy may also cause electrolyte alterations: platinum compounds can induce sodium, potassium, and magnesium alterations, and alkylating agents and vinca alkaloids are usually associated with SIADH [13].

## 2. Open Issues

Despite the number of studies on the endocrine toxicity of antineoplastic therapy, many areas of uncertainty remain. First, the pathogenesis of endocrine side effects, especially for TKIs, is not always clear [8,10], and the possible association of different treatments could complicate the clinical presentation and management [29]. Second, endocrine toxicities vary between patients, suggesting that other factors such as pre-existing diseases or genetic aspects can play a role [19], and the comprehension of these confounding factors is necessary. Third, some authors have stated that the Common Terminology Criteria for Adverse Events scale is not very reliable for endocrine toxicities, since the differentiation between grades 2 and 3 is difficult, although necessary, since the clinical approaches are very different [8,30]. Finally, the increase in life expectancy of patients affected by cancer could highlight long-term complications of these treatments, as partially described for cancer survivors.

## 3. Future Directions: Towards an Oncological Endocrinologist

Considering what has been reported so far, endocrine complications are a clinically significant problem for patients suffering from cancer for two main reasons: the clinical impact of endocrine toxicities, which can be life-threatening and can strongly impair quality of life, and the possible antineoplastic treatment discontinuation, frequent in the case of incorrect or late management of endocrine toxicities.

It is therefore necessary, especially in specialized oncological centers, to include the figure of the endocrinologist with a precise expertise in endocrine toxicities of antineoplastic therapy and to establish a strong collaboration between endocrinologists and oncologists in order to increase awareness on these issues and optimize oncological treatment.

## Abbreviations

**TKI**	tyrosine kinase inhibitor
**mTOR**	mammalian targets of rapamycin
**TSH**	thyrotropin-stimulating hormone
**SIADH**	syndrome of inappropriate antidiuretic hormone secretion
**PD-1**	programmed cell death protein 1
**PD-L1**	programmed cell death protein ligand 1
**CTLA-4**	cytotoxic T-lymphocyte antigen 4
**ACTH**	adrenocorticotropic hormone

## References

[1] Tang J., Shalabi A., Hubbard-Lucey V.M. (2018). Comprehensive analysis of the clinical immuno-oncology landscape. Ann. Oncol..

[2] Ruggeri R.M., Campennì A., Giuffrida G., Trimboli P., Giovanella L., Trimarchi F., Cannavò S. (2018). Endocrine and metabolic adverse effects of immune checkpoint inhibitors: An overview (what endocrinologists should know). J. Endocrinol. Investig..

[3] Krause D.S., Van Etten R.A. (2005). Tyrosine Kinases as Targets for Cancer Therapy. N. Engl. J. Med..

[4] Gabora K., Piciu A., Bădulescu I.C., Larg M.I., Stoian I.-A., Piciu D. (2019). Current evidence on thyroid related adverse events in patients treated with protein tyrosine kinase inhibitors. Drug Metab. Rev..

[5] Lodish M.B. (2013). Clinical review: Kinase inhibitors: Adverse effects related to the endocrine system. J. Clin. Endocrinol. Metab..

[6] Jannin A., Penel N., Ladsous M., Vantyghem M.C., Cao C.D. (2019). Tyrosine kinase inhibitors and immune checkpoint inhibitors-induced thyroid disorders. Crit. Rev. Oncol. Hematol..

[7] Rizza L., Sbardella E., Gianfrilli D., Lauretta R., Tenuta M., Del Bene G., Longo F., Faggiano A., Lenzi A., Giannetta E. (2020). Thyroid profile during the alternative Sunitinib dosing 2/1 schedule in metastatic renal cell carcinoma. Endocrine.

[8] Castinetti F., Albarel F., Archambeaud F., Bertherat J., Bouillet B., Buffier P., Briet C., Cariou B., Caron P., Chabre O. (2018). Endocrine side-effects of new anticancer therapies: Overall monitoring and conclusions. Ann. d’Endocrinol..

[9] Fallahi P., Ferrari S.M., Elia G., Ragusa F., Paparo S.R., Camastra S., Mazzi V., Miccoli M., Benvenga S., Antonelli A. (2020). Therapy of Endocrine Disease: Endocrine-metabolic effects of treatment with multikinase inhibitors. Eur. J. Endocrinol..

[10] Buffier P., Bouillet B., Smati S., Archambeaud F., Cariou B., Verges B. (2018). Expert opinion on the metabolic complications of new anticancer therapies: Tyrosine kinase inhibitors. Ann. d’Endocrinol..

[11] Gallo M., Muscogiuri G., Felicetti F., Faggiano A., Trimarchi F., Arvat E., Vigneri R., Colao A. (2018). Adverse glycaemic effects of cancer therapy: Indications for a rational approach to cancer patients with diabetes. Metabolism.

[12] Vergès B., Walter T., Cariou B. (2014). ENDOCRINE SIDE EFFECTS OF ANTI-CANCER DRUGS: Effects of anti-cancer targeted therapies on lipid and glucose metabolism. Eur. J. Endocrinol..

[13] Verzicco I., Regolisti G., Quaini F., Bocchi P., Brusasco I., Ferrari M., Passeri G., Cannone V., Coghi P., Fiaccadori E. (2020). Electrolyte Disorders Induced by Antineoplastic Drugs. Front. Oncol..

[14] O’Sullivan S., Lin J.-M., Watson M., Callon K.E., Tong P.C., Naot D., Horne A., Aati O., Porteous F., Gamble G. (2011). The skeletal effects of the tyrosine kinase inhibitor nilotinib. Bone.

[15] Berman E., Nicolaides M., Maki R.G., Fleisher M., Chanel S., Scheu K., Wilson B.-A., Heller G., Sauter N.P. (2006). Altered Bone and Mineral Metabolism in Patients Receiving Imatinib Mesylate. N. Engl. J. Med..

[16] Schumacher T.N., Schreiber R.D. (2015). Neoantigens in cancer immunotherapy. Science.

[17] Gajewski T.F., Schreiber H., Fu Y.-X. (2013). Innate and adaptive immune cells in the tumor microenvironment. Nat. Immunol..

[18] Gong J., Chehrazi-Raffle A., Reddi S., Salgia R. (2018). Development of PD-1 and PD-L1 inhibitors as a form of cancer immunotherapy: A comprehensive review of registration trials and future considerations. J. Immunother. Cancer.

[19] Yang H., Yao Z., Zhou X., Zhang W., Zhang X., Zhang F. (2020). Immune-related adverse events of checkpoint inhibitors: Insights into immunological dysregulation. Clin. Immunol..

[20] Barroso-Sousa R., Barry W.T., Garrido-Castro A.C., Hodi F.S., Min L., Krop I.E., Tolaney S.M. (2018). Incidence of Endocrine Dysfunction Following the Use of Different Immune Checkpoint Inhibitor Regimens: A Systematic Review and Meta-analysis. JAMA Oncol..

[21] Barnabei A., Carpano S., Chiefari A., Bianchini M., Lauretta R., Mormando M., Puliani G., Paoletti G., Appetecchia M., Torino F. (2020). Case Report: Ipilimumab-Induced Panhypophysitis: An Infrequent Occurrence and Literature Review. Front Oncol..

[22] Haanen J., Carbonnel F., Robert C., Kerr K., Peters S., Larkin J., Jordan K., Committee E.G. (2017). Management of toxicities from immunotherapy: ESMO Clinical Practice Guidelines for diagnosis, treatment and follow-up. Ann. Oncol..

[23] Faje A. (2016). Immunotherapy and hypophysitis: Clinical presentation, treatment, and biologic insights. Pituitary.

[24] Torino F., Barnabei A., De Vecchis L., Appetecchia M., Strigari L., Corsello S.M. (2012). Recognizing menopause in women with amenorrhea induced by cytotoxic chemotherapy for endocrine-responsive early breast cancer. Endocr. Relat. Cancer.

[25] Oktay K., Harvey B.E., Partridge A.H., Quinn G.P., Reinecke J., Taylor H.S., Wallace W.H., Wang E.T., Loren A.W. (2018). Fertility Preservation in Patients With Cancer: ASCO Clinical Practice Guideline Update. J. Clin. Oncol..

[26] Candy B., Jones L., Vickerstaff V., Tookman A., King M. (2016). Interventions for sexual dysfunction following treatments for cancer in women. Cochrane Database Syst. Rev..

[27] Schover L. (2018). Sexual quality of life in men and women after cancer. Climacteric.

[28] Handforth C., D’Oronzo S., Coleman R.E., Brown J.E. (2018). Cancer Treatment and Bone Health. Calcif. Tissue Int..

[29] Larkin J., Chiarion-Sileni V., Gonzalez R., Grob J.J., Cowey C.L., Lao C.D., Schadendorf D., Dummer R., Smylie M., Rutkowski P. (2015). Combined nivolumab and ipilimumab or monotherapy in untreated melanoma. N. Engl. J. Med..

[30] Illouz F., Briet C., Cloix L., Le Corre Y., Baize N., Urban T., Martin L., Rodien P. (2017). Endocrine toxicity of immune checkpoint inhibitors: Essential crosstalk between endocrinologists and oncologists. Cancer Med..

